# Nattokinase, a Subtilisin-like Alkaline-Serine Protease, Reduces Mutacin Activity by Inactivating the Competence-Stimulating Peptide in *Streptococcus mutans*

**DOI:** 10.3390/pathogens13040286

**Published:** 2024-03-27

**Authors:** Manami Kimijima, Naoki Narisawa, Eiji Hori, Kengo Mandokoro, Tatsuro Ito, Yukina Ota, Momoko Sashida, Yasushi Kawai, Fumio Takenaga

**Affiliations:** 1 Bioresource Utilization Sciences, Nihon University Graduate School of Bioresource Sciences, Fujisawa 252-0880, Kanagawa, Japan; brma22502@g.nihon-u.ac.jp (M.K.);; 2 Department of Pediatric Dentistry, Nihon University School of Dentistry at Matsudo, Matsudo 271-8587, Chiba, Japan; 3 Research Institute of Oral Science, Nihon University School of Dentistry at Matsudo, Matsudo 271-8587, Chiba, Japan; 4 Nihon University Graduate School of Dentistry at Matsudo, Pediatric Dentistry, Matsudo 271-8587, Chiba, Japan

**Keywords:** *Streptococcus mutans*, mutacin, caries, competence-stimulating peptide, competence, bacteriocin, nattokinase

## Abstract

*Streptococcus mutans* is a major cariogenic organism because of its ability to form biofilms on tooth surfaces. Bacteriocins produced by *S. mutans* (known as mutacins) are indirect pathogenic factors that play a role in the persistence of this microbe in the oral environment. Nattokinase, a subtilisin-like alkaline serine protease, potently inhibits biofilm formation without affecting *S. mutans* growth. However, effective strategies utilizing nattokinase to control mutacin production by *S. mutans* are lacking. In this study, we evaluated the effect of nattokinase on mutacin activity in 46 strains of *S. mutans* with different mutacin genotypes isolated from the dental plaques of pediatric patients with caries. Nattokinase reduced the activity of mutacin against oral streptococci at a concentration of 1 mg/mL in all clinical isolates. Furthermore, nattokinase reduced the expression of non-lantibiotic mutacin structural genes (*nlmABCD*) and inactivated the extracellular competence-stimulating peptide involved in *comDE* activation, which regulates non-lantibiotic mutacin gene expression. These results suggest that nattokinase may reduce the virulence of *S. mutans* and could potentially be used as a new caries-preventive agent as an alternative to conventional drug treatments.

## 1. Introduction

Dental caries is a bacterial infection resulting from demineralization of tooth enamel by acids derived from oral microorganisms. The global economic burden of treating dental caries amounts to billions of dollars annually. *Streptococcus mutans* is considered a major cariogenic organism owing to its ability to form biofilms on tooth surfaces and because of its acid tolerance and production. Bacteriocin production is an indirect virulence factor in *S. mutans*. The bacteriocin produced by *S. mutans* was first described by Kuramitsu et al. as “mutacin” [1]. We also refer to *S. mutans* bacteriocins as “mutacin” in this paper. Mutacins help *S. mutans* compete with other streptococci during the early stages of dental biofilm formation to establish or maintain colonization on the tooth surface [1]. Therefore, mutacin is an attractive target for reducing *S. mutans*-related infections.

Two types of mutacins have been characterized; these are lantibiotic and non-lantibiotic mutacins. At least five lantibiotic and three non-lantibiotic mutacins have been reported [2], with *S. mutans* possessing one or more of these genes [3,4]. In general, lantibiotics have a broader spectrum of antimicrobial activities than non-lantibiotics. Non-lantibiotic mutacins are highly conserved among *S. mutans,* play an important role in intra- and interspecies interactions in dental biofilms [2], and are controlled by the activation of the two-component ComCDE system. The ComCDE system is activated by an optimal extracellular concentration of a signaling molecule called competence-stimulating peptide (CSP) [5,6]. Optimal extracellular concentrations of CSP induce autophosphorylation of the sensor kinase ComD and subsequent phosphorylation of the response regulator ComE. Phosphorylated ComE activates gene expression via the mutacin promoter, resulting in a substantial increase in mutacin production. Accordingly, disruption of the ComCDE system may play an important role in the regulation of mutacins. Although the importance of mutacin production in pathogenicity and the mechanisms of mutacin production have been widely researched, effective strategies for controlling mutacin production are lacking.

We previously found that natto, an unsalted fermented soybean food produced using *Bacillus subtilis* var. natto as a starter, inhibited *S. mutans* biofilm formation, and the inhibitory factor was nattokinase (subtilisin; EC 3.4.21.62) [7,8], which is a major extracellular enzyme produced by *B. subtilis* var. natto [9] and a subtilisin-like alkaline serine protease that exhibits potent thrombolytic activity and substrate specificity. Nattokinase did not show growth-inhibiting or bactericidal activity against *S. mutans* or other commensal bacteria in the oral cavity and inhibited the synthesis of insoluble glucan, the major component of the extracellular matrix of *S. mutans* [7,8]. Owing to its natural occurrence, safety, efficiency, and cost-effectiveness, this enzyme is considered a potential caries-preventive agent. However, the effect of nattokinase on mutacin activity has not yet been determined. The purpose of this study is to clarify the inhibitory effect of nattokinase on mutacin activity against *S. mutans* with different mutacin genotypes isolated from the human oral cavity and its inhibitory mechanism.

## 2. Materials and Methods

### 2.1. Bacterial Strains and Culture Conditions

Brain–heart infusion (BHI) medium (Becton Dickinson and Company, San Jose, CA, USA) was used to maintain the bacterial culture and perform the mutacin assay (Section 2.7 and Section 2.11). Mitis–salivarius medium (Becton Dickinson and Company) containing 1.5% (*w*/*v*) agar was used for the isolation of *S. mutans*. *S. mutans* UA159 (ATCC 700610), *S. mutans* NBRC13955, *S. mutans* clinical isolates, *Streptococcus gordonii* DL1, *Streptococcus sanguinis* ATCC10556, *Streptococcus mitis* ATCC6249, *Streptococcus salivarius* ATCC9759, and *Streptococcus sobrinus* 6715 were cultured at 37 °C in a 5% CO_2_ incubator. 

### 2.2. Isolation and Identification of S. mutans

Seventeen children (aged 3–9 years) participated in this study (ethics committee approval no. EC20-28; Nihon University School of Dentistry at Matsudo, Chiba, Japan). The decayed, missing, and filled tooth indices of these patients ranged from 4 to 16. Plaque was collected from the tooth surface using a sterile excavator, suspended in 1 mL of 0.9% (*w*/*v*) NaCl solution, applied to mitis–salivarius agar, and incubated at 37 °C for 48 h. Rough colonies, characteristic of *S. mutans*, were isolated. Species in the genus were identified based on the presence or absence of a portion of the *htrA* gene encoding a surface protease and an intergenic region following polymerase chain reaction (PCR) amplification, as reported by Chen et al. [10]. Genomic DNA was extracted from the culture medium using ISOPLANT (Nippon Gene Co., Ltd., Tokyo, Japan) following the manufacturer’s protocols. The PCR mixture was prepared by mixing 6 µL PrimeSTAR Max Premix (2×) (Takara Bio Inc., Shiga, Japan), 10 ng genomic DNA, forward (5′-TCGCGAAAAAGATAAACAAACA-3′) and reverse (5′-GCCCCTTCACAGTTGGTTAG-3′) primers (final concentration of 0.2 µM each), and PCR-grade water. The PCR conditions were as follows: 30 cycles at 98 °C for 10 s, 55 °C for 10 s, and 72 °C for 6 s. The PCR products were subjected to MultiNA capillary electrophoresis (MCE-202; Shimadzu Corporation, Kyoto, Japan) to confirm amplification.

### 2.3. Confirmation of Serotypes

Serotypes *c, e, f*, and *k* were confirmed via PCR using serotype-specific sets of primers as described previously [11]. The composition of the PCR mixture was as follows: 5 µL Premix Taq™ (TaKaRa Taq™ Version 2.0), 5 ng DNA, and 5 µM primers, scaled to 10 µL with PCR-grade water. The PCR was performed according to a previously reported method [11]. PCR products were confirmed using the MultiNA capillary electrophoresis system (Shimadzu Corporation).

### 2.4. Arbitrarily Primed PCR

PCR was performed using OPA-02 (5’-TGCCGAGCTG-3’). The composition of the PCR mixture was as follows: 5 µL Premix Taq™ (TaKaRa Taq™ Version 2.0), 5 ng DNA, and 5 µM primers, scaled to 10 µL with PCR-grade water. The PCR was performed according to a previously reported method [12]. The PCR products were confirmed using the MCE-202 MultiNA capillary electrophoresis system. All amplification reactions were independently repeated at least thrice to obtain reproducible, accurate, and distinct banding patterns. Genotypes were scored as “0” or “1” depending on the absence or presence of bands, respectively. Phylogenetic trees were constructed using the statistical analysis software “R” (version 3.4.3), based on the unweighted pair group method with arithmetic mean (UPGMA method).

### 2.5. PCR Screening of Mutacin Genes

The primers used are listed in Table S1. The composition of the PCR reaction solution was as follows: 5 µL EmeraldAmp^®^ MAX PCR Master Mix (2× Premix) (Takara Bio Inc., Shiga, Japan), 25 ng genomic DNA, and forward and reverse primers (final concentration 2.5 µM each); the total volume was made up to 10 µL with PCR-grade water. PCR conditions were as follows: 30 cycles of denaturation for 1 min at 98 °C, annealing for 1 min at 58 °C, and extension for 1 min at 70 °C. The PCR products were electrophoresed on a 2% (*w*/*v*) agarose gel for 30 min at 100 V, and amplification was confirmed by the presence of bands after staining with ethidium bromide.

### 2.6. Nattokinase

Nattokinase (E.C. 3.4.21.62) (Fujifilm Wako Pure Chemicals, Osaka, Japan) was dissolved in sterile distilled water. The solution was sterilized via filtration (0.22 μm cellulase acetate filter; Sartorius, Göttingen, Germany) and appropriately diluted in distilled water. Nattokinase solution was stored at 4 °C for 5 d or at −30 °C for a long duration.

### 2.7. Mutacin Assay

Mutacin production by *S. mutans* was determined using a modified version of the method described by Tamura et al. [13]. Overnight cultures of *S. mutans* were punctured onto BHI 1.5% (*w*/*v*) agar plates and incubated at 37 °C in a 5% CO_2_ atmosphere for 20 h. The plates were overlaid with BHI agar containing 1% (*v*/*v*) indicator strain culture. After layering, the plates were incubated at 37 °C in a 5% CO_2_ atmosphere for 2 d, and the inhibition size against the indicator strain was determined. Data represent the average values from three independent experiments.

### 2.8. RNA Extraction 

RNA was extracted using ISOGEN RNA Extraction Reagent (Nippon Gene Co., Ltd.) according to the manufacturer’s instructions. Aliquots (1 mL) of *S. mutans* UA159 overnight culture were centrifuged at 11,200× *g* for 5 min, and the pellet was suspended in 1 mL sterile water to prepare the bacterial solution. Aliquots (10 mL) of BHI medium were inoculated with 10% (*v*/*v*) bacterial solution and incubated at 37 °C in a 5% CO_2_ atmosphere until mid-log phase (OD_600_ = 0.45 to 0.50). The culture was centrifuged at 11,200× *g* for 5 min at 4 °C, and the resulting bacterial pellet was dissolved in 10 mL of fresh BHI medium to obtain a final concentration of 1 mg/mL nattokinase. The bacterial cells were incubated for 10 min at 37 °C. After incubation, the culture was centrifuged at 11,200× *g* at 4 °C for 15 min, and the resulting bacterial pellet was dissolved in 0.3 mL of ISOGEN solution. The sample was transferred to a 2 mL screw-capped tube, an appropriate amount of zirconia beads (Ф0.5 mm zirconia/silica beads, BioSpec Products, Bartlesville, OK, USA) was added, and the sample was processed in a bead crusher (µT-12; TAITEC Co., Saitama, Japan) at 3200 rpm for 180 s. The RNA-containing supernatant was treated with 700 μL ISOGEN for 30 min at 55 °C. After centrifugation, total RNA was subjected to chloroform, isopropanol, and ethanol precipitation for purification. The resulting RNA was dissolved in RNase-free water and stored at −80 °C.

### 2.9. Quantitative Reverse Transcription-PCR

cDNA was synthesized using a PrimeScript™ FAST RT reagent kit with gDNA Eraser (Takara Bio Inc.). qRT-PCR was performed using the TB Green™ Premix Ex Taq™ II (Tli RNaseH Plus) kit (Takara Bio Inc.) and the Step One Plus Real-Time PCR System (Applied Biosystems, Foster City, CA, USA). The volume of the reaction mixture was set to 10 μL (5 μL 2× TB Green Premix Ex Taq II, 0.2 μL 50× ROX Reference Dye, 1 μL of 10 ng/µL cDNA, 0.2 μL forward primer, 0.2 μL reverse primer, and 3.4 μL PCR-grade water). The reaction conditions were as follows: one cycle of 3 min at 95 °C, followed by 40 cycles of 5 s at 95 °C and 34 s at 60 °C. Gene expression was normalized with that of the reference *16S* rRNA gene using the 2^−ΔΔCT^ method. The primers used are listed in Table S1. Data represent the average values from three independent experiments.

### 2.10. Construction of comC Mutant

The *comC* mutant strain of *S. mutans* UA159 was generated via double homologous recombination of the erythromycin resistance gene. Sequence information was obtained from the *S. mutans* UA159 genome (GenBank accession number: NC_004350.2). The 5′ flanking region of the *comC* gene was amplified from genomic DNA via PCR. The amplified fragment was inserted into the EcoRI-KpnI site of the cloning vector pUC19 (Takara Bio Inc.) [14]. Next, a PCR-amplified fragment of the 3′ flanking region of the *comC* gene was inserted into the XbaI-HindIII region of pUC19 with 5’ flanking region of the *comC* gene. The resulting plasmid was digested with BamHI, and the erythromycin resistance gene from pResEmMCS10 [15] was introduced. The resultant plasmid, pREScomC, was linearized via digestion with EcoRI and HindIII and cloned into *S. mutans* using a previously reported method [16]; 10% (*v*/*v*) of the UA159 strain preculture was mixed with 10% (*v*/*v*) heat-treated (90 °C for 15 min) Gibco^®^ horse serum (Thermo Fisher Scientific, Rocklin, CA, USA) in BHI medium. Cells were incubated at 37 °C in a 5% CO_2_ atmosphere until reaching the mid-log phase and used as competent cells. Linearized DNA fragments were added to the competent cells and cultured for an additional 1.5 h; the cells were then plated on BHI agar containing erythromycin (Sigma-Aldrich, St. Louis, MO, USA) at a final concentration of 10 µg/mL at 37 °C in a 5% CO_2_ atmosphere for 2 d to obtain erythromycin-resistant strains. The transformants were assessed for mutations via sequencing.

### 2.11. Effect of Nattokinase on CSP

CSP (SGSLSTFFRLFNRSFTQALGK) was chemically synthesized (Eurofins Genomics, Inc., Tokyo, Japan). The synthesized CSP was dissolved in sterile water and diluted to an appropriate concentration. Aliquots (9 µL) of CSP (1 mg/mL) were mixed with 1 µL nattokinase (10 mg/mL) and incubated at 37 °C for 1 h. Aliquots (5 µL) of the reaction solution were mixed with 5 µL of an overnight bacterial culture at 37 °C in a 5% CO_2_ atmosphere and punctured on a BHI agar plate. The plates were then covered with 10 mL agar medium containing 5% (*v*/*v*) of an *S. gordonii* overnight culture and incubated at 37 °C in a 5% CO_2_ atmosphere for 20 h_._ The diameter of the resulting inhibition zones was measured. Data are expressed as the mean ± standard deviation of values from three independent experiments.

## 3. Results

### 3.1. Characterization of the Clinical Isolate

We isolated a total of 46 *S. mutans* strains from the plaques of 17 pediatric patients with dental caries. To understand the characteristics of the isolates, their serotypes and genotypes were determined (Table 1). Based on known PCR-based serotyping, 38 strains obtained in this study were type c, six strains were type e, and two strains were type c/k. Arbitrarily primed PCR (AP-PCR) has been widely used to study the genotypic similarities and variations in *S. mutans* [12]. AP-PCR was used to evaluate the genotypic differences among *S. mutans* strains. The OPA-02 primer resulted in robust amplification, and 17 distinct bands within 250–4000 bp were obtained (Figure S1). A phylogenetic tree generated on the basis of reproducible banding patterns showed that 46 strains were classified into 23 genotypes (Figure S2). The laboratory strains UA159 and NBRC13955 were classified as AP-PCR genotypes 9 and 12, respectively.

Genomic information revealed that the laboratory strains UA159 and NBRC13955 used in this study possessed three mutacin structural genes—*nlmAB* (mutacin IV), *nlmC* (mutacin V), and *nlmD* (mutacin VI). All 46 strains contained multiple mutacin structural genes, as detected using PCR amplification (Table 2). All strains were *mutA* (mutacin I, II, and III) negative and *nlmC* positive. Fifteen strains were *mukA* (mutacin K8) positive, 21 were *smbAB* (smb) positive, 25 were *nlmAB* positive, and 44 were *nlmD* positive. Based on the pattern of mutacin structural gene possession in the 46 isolates used in this study, they were classified into nine genotypes (Table 1 and Table 2). Of these, mutacin genotype 4 *(nlmABCD* positive) was the most common, accounting for approximately 30% of the total number of strains. The laboratory strains UA159 and NBRC13955 were classified as mutacin genotype 4 strains. No association was found among the AP-PCR genotype, mutacin genotype, and serotype (Table 1 and Figure S2).

The mutacin activity of *S. mutans* against five different oral streptococcal strains was assessed based on the size of the inhibition zone. The results are summarized in Table 1. All genotypes showed inhibition against *S. sanguinis*, *S. gordonii*, *S. mitis*, and *S. salivarius*; however, inhibition against *S. sobrinus* was rather weak. Large differences in inhibition were observed, even among strains of the same genotype. Clinical isolates had two to five mutacin structural genes (Table 1). However, no correlation was observed between mutacin activity and the number of structural genes in *S. mutans*.

### 3.2. Effect of Nattokinase on Mutacin Activity 

All clinical *S. mutans* strains showed a reduction in biofilm biomass by approximately 50% or more at a nattokinase concentration of 1.0 mg/mL when biofilm formation was evaluated using the microtiter plate method (Figure S3). The amount of biofilm formation by UA159 was not affected by the addition of heat-treated (90 °C, 15 min) nattokinase at 1.0 mg/mL. Based on these results, the effect of a 1.0 mg/mL concentration of nattokinase on mutacin activity was evaluated. Table 3 shows the mutacin activity in the presence of nattokinase. The heat-inactivated nattokinase at 1.0 mg/mL had no effect on mutacin activity. All *S. mutans* strains showed reduced mutacin activity in the presence of nattokinase—29 strains showed decreased activity against *S. sanguinis*, 42 strains showed decreased activity against *S. gordonii*, 25 strains showed decreased activity against *S. mitis*, 11 strains showed decreased activity against *S. sobrinus*, and 32 strains showed decreased activity against *S. salivarius*.

Almost all *S. mutans* strains obtained in this study possessed *nlmC* and *nlmD* (Table 1). The mutacins encoded by these two genes lack antimicrobial activities [3]. Therefore, we evaluated the association between the presence of *nlmAB*, *mukA,* and *smbAB* and the sensitivity to nattokinase (Table 4). Nattokinase had a strong inhibitory effect on mutacin activity against strains positive for only *nlmAB* and those positive for both *nlmAB* and *smbAB*. In contrast, nattokinase had only a weak inhibitory effect against strains positive for *mukA*. No association was found between the number of mutacin genes and susceptibility to nattokinase.

### 3.3. Effect of Nattokinase on Mutacin Structural Gene Expression

**Figure 1:**
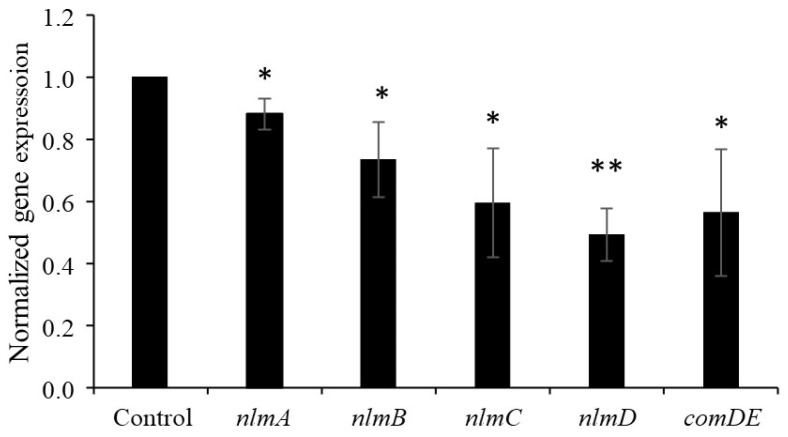
**Effect of nattokinase on gene expression in *S. mutans* UA159.** Results are expressed as relative values, with the control set as “1”. Data are expressed as the mean ± standard deviation of values from three independent experiments. Different letters indicate significant differences based on standard *t*-test (* *p* < 0.05, ** *p* < 0.01).

**Figure 2:**
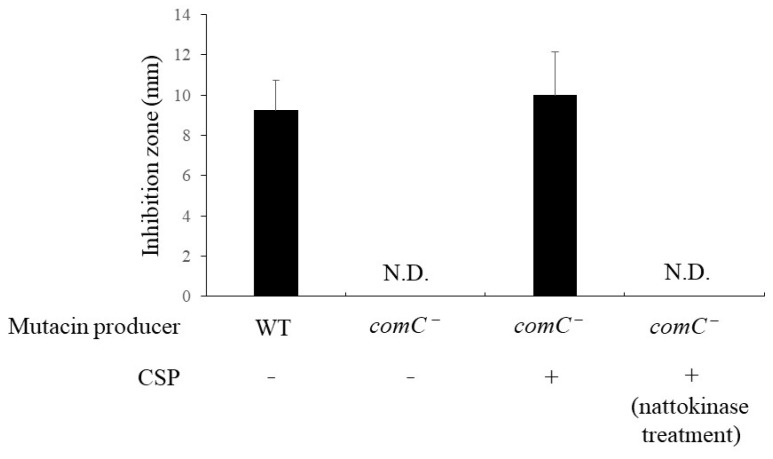
**Effect of nattokinase on mutacin production in *S. mutans* UA159 *comC* mutant.** Artificial CSP was added at a final concentration of 80 µM. *S. gordonii* DL1 was used as an indicator strain. CSP was treated with nattokinase for 1 h at 37 °C. ‘WT’ and ‘*comC*^−^’ of mutacin-producing bacteria represent wild types and *comC*^−^ mutants, respectively. ‘N.D.’ indicates not detected. The average diameter (mm) is expressed as the mean ± standard deviation of values from three independent experiments. “N.D.” indicates “not detected”; CSP, competence-stimulating peptide.

We evaluated the effect of nattokinase on the expression of structural mutacin genes. Nattokinase was added to cells in the logarithmic growth phase, and the cells were analyzed after incubation for 10 min. The results showed that the expression of *nlmABCD*, a structural gene of mutacins IV, V, and VI, decreased in the presence of 1 mg/mL nattokinase (Figure 1). The expression of *nlmABCD* is regulated by the ComCDE system [2]. The *comC* gene synthesizes pre-CSP, which consists of 21 amino acid residues. Pre-CSP is modified into an activated form, resulting in the activation of ComDE [17]. The *comDE* gene was downregulated by nattokinase (Figure 1). This result was attributed to the nattokinase-mediated inhibition of CSP. To confirm this hypothesis, a bioassay was performed using the *comC* mutant strain. The *comC* mutant completely lost its mutacin activity against *S. gordonii*, and the addition of artificially synthesized CSP restored its mutacin activity (Figure 2). The *comC* mutant did not show recovery of mutacin activity upon the addition of nattokinase-treated CSP (Figure 2). The parent strain formed an 8 mm inhibition zone against *S. gordonii* when spotted with untreated and heat-inactivated nattokinase-treated CSP. The parent strain also showed an 8 mm inhibition zone when spotted with nattokinase-treated CSP. This indicated that nattokinase inhibited mutacin synthesis via CSP inhibition. In a spot assay conducted on agar medium, 80 µM CSP had no effect on the growth of *comC* mutant (Figure S4).

## 4. Discussion

Phenotypic and genotypic diversity can increase population stability and productivity through cooperative interactions among community members [18,19]. All *S. mutans* clinical isolates obtained in this study possessed multiple mutacin synthesis genes and showed diverse possession patterns. This suggests that mutacins play an important role in the acquisition of niche-related mutations by *S. mutans* in the oral environment. One factor that contributes to the emergence of diversity is genome modification through the acquisition of exogenous DNA. Natural competence refers to the ability of prokaryotes to actively acquire and internalize extracellular DNA from the environment. The ComCDE system in *S. mutans* induces competent mutacin gene expression [2]. Activation of the ComCDE system is tightly regulated in response to extracellular CSP concentration. Kreth et al. showed that competence induction can result in interspecies transfer of transformed DNA from *S. gordonii*, an endemic oral bacterium, to *S. mutans* [20,21]. This gene transfer phenomenon is dependent on the activity of mutacin IV in *S. mutans*. Based on these results, Merritt and Qi [2] described a possible new ecological role of mutacin in transforming DNA from closely related organisms that can be used for genome repair or to acquire new fitness-enhancing traits. Dufour et al. [22] also showed that mutacin V may play a role as a peptide regulator in the transcriptional control of competence regulons. In the present study, we found that nattokinase inactivated CSP and downregulated *comDE*. Continuous exposure of nattokinase to *S. mutans* may not only reduce mutacin productivity but also suppress the emergence of genetic diversity and thus environmental adaptability. Inhibition of the ComCDE system by nattokinase is adaptable to other proteases [23,24].

Nattokinase is derived from *B. subtilis* var. natto. Fermented soybean natto produced using this strain as a starter contains abundant proteases, among which nattokinase is the major one [25]. The protease activity at the concentration of nattokinase used in this study (final concentration 1 mg/mL) was equivalent to that of 10 g wet weight of natto product [8]. Nattokinase is essentially safe when administered at the recommended dose (200 mg/d); histopathological examination of the organs and tissues have shown no evidence of toxicity [9]. Approximately 40 g of natto is consumed per serving. Accordingly, the regular consumption of natto is expected to reduce the virulence of *S. mutans*. 

To reduce the risk of dental caries development, safe functional foods and their ingredients have been researched and developed. However, the consumption of foods/beverages is considered to result in insufficient contact time between the oral biofilm and the active ingredient. The observation that nattokinase reduced the expression of mutacin structural genes is considered an important finding for clinical applications. In the future, it may be necessary to develop a method to utilize nattokinase in toothpaste and tablets obtained via nonthermal processing. In addition, the use of probiotics with high enzyme productivity could offer a solution to achieve the full effect of proteases. Serine-type proteases from *S. gordonii*, an oral bacterium, have been shown to reduce the levels of virulence factors in *S. mutans* [26]. Furthermore, *B. subtilis* var. natto produces more enzymes than *B. subtilis*. Therefore, *B. subtilis* var. natto may be beneficial as a probiotic.

Bacteriocins, produced by lactic acid bacteria, are peptides or proteins with antimicrobial activities. It is reasonable to assume that nattokinase with protease activity can also hydrolyze mutacins. A 20-fold concentrated crude mutacin fraction from 30 mL *S. mutans* UA159 culture formed a 10 mm inhibition zone against *S. gordonii* at a volume of 50 µL (Figure S5). The purified fraction lost its inhibitory activity against *S. gordonii* when allowed to react with 1 mg/mL nattokinase. Five clinical isolates were selected according to differences in mutacin genotype and mutacin activity. Similar results were observed for mutacins derived from C5-1, C10-1, C8-1, C16-1, and C17-1. This suggests that nattokinase exhibits hydrolytic activity against different types of mutacins. Future evaluation of the effect of nattokinase on purified mutacin is important for controlling *S. mutans*. Nattokinase was highly effective in inhibiting mutacin activity against various *S. mutans* strains but had a limited effect against *muk*-positive strains. The *mukA* gene is presumably regulated by the two-component MukRK system (SMU. 1814–1815) at the same locus [27]; however, the details of the regulatory system for gene expression have not been elucidated. The detailed mechanism underlying the effect of nattokinase on mutacin K8 activity requires further investigation.

Large differences in inhibition were observed, even between strains of the same genotype (Figure S1). Similar results were reported by Watanabe et al. [3] and were explained by the presence of unknown mutacins and altered activity due to mutational changes in known genes. The presence of new mutacins and the efficacy of nattokinase against these mutacins could not be determined in this study.

Mutacin transcription is highly dependent on extremely high-cell-density growth conditions, such as those in biofilms. As mentioned above, nattokinase effectively inhibits *S. mutans* biofilm formation. Therefore, nattokinase may potentially deprive the mutacin-producing environment. As nattokinase exhibited no bactericidal activity against *S. mutans* or other oral commensal bacteria, it was thought that damage to the oral commensal flora would be minimal. Nattokinase does not produce resistant bacteria, and its safety is ensured. Nattokinase is expected to be used as a new oral health agent. Clinical evaluation of the efficacy of nattokinase was not possible in this study. Future clinical evaluation of its potential in humans is required.

## 5. Conclusions

Nattokinase is a subtilisin-like alkaline serine protease from *B. subtilis* var. natto and is abundant in fermented natto products. *S. mutans* produces a variety of mutacins that contribute to oral colonization. This was the first report to clarify that nattokinase decreases the mutacin activity of *S. mutans*. This appears to prevent the production of bacteriocin by inactivating CSP by nattokinase, thereby reducing the expression of the mutacin synthesis gene in *S. mutans*. In addition, this is due to direct hydrolysis of mutacin by nattokinase. This suggests that nattokinase suppresses mutacin productivity. Our group has previously shown that nattokinase suppresses biofilm formation in *S. mutans*. These results suggest that nattokinase may be a new caries-preventive agent.

## Figures and Tables

**Table 1:** Characterization of 46 clinical isolates and 2 laboratory strains.

Patient No.	Isolation No.	Serotype	AP-PCR Genotype	Mutacin Genotype	Indicator Strains (mm)				
					*S. sanguinis*	*S. gordonii*	*S. mitis*	*S. sobrinus*	*S. salivarius*
C1	1	c	17	4	12.33	10.33	3.67	N.D.	7.00
	2	c	17	4	2.00	4.33	3.33	N.D.	3.33
	3	c	17	4	1.33	4.33	3.50	N.D.	3.33
C2	1	c	20	4	3.67	4.33	3.50	N.D.	4.00
	2	c	11	4	3.33	4.33	3.00	1.00	3.67
	3	c	18	4	3.33	4.33	3.00	N.D.	3.33
C3	1	e	10	4	2.33	4.33	3.33	N.D.	5.00
	2	e	10	4	N.D.	4.67	3.50	N.D.	4.33
	3	e	10	4	1.00	4.00	3.33	N.D.	7.67
C4	1	c	14	6	21.67	15.00	6.67	4.33	12.00
	2	c	13	6	21.00	13.33	11.33	6.67	11.33
	3	c	13	6	19.33	12.67	9.33	6.67	12.00
C5	1	c	10	3	11.67	11.67	4.67	1.00	10.67
	2	c	10	3	13.00	14.00	4.00	N.D.	12.00
	3	c	10	3	14.33	11.67	3.00	N.D.	9.00
C6	1	c	16	1	2.33	4.00	3.67	N.D.	3.33
C7	1	c	15	3	2.33	4.00	4.33	1.33	3.33
	2	c	12	3	1.00	3.67	2.00	N.D.	2.00
	3	c	12	3	N.D.	3.67	N.D.	N.D.	3.33
C8	1	c	22	2	8.33	9.33	8.67	6.00	10.00
	2	c	22	9	12.67	10.00	8.33	4.00	9.67
	3	c	22	9	13.67	10.67	9.33	4.67	10.00
C9	1	c	4	4	0.00	6.00	2.33	N.D.	4.00
	2	c	4	4	0.00	6.00	2.00	N.D.	4.67
	3	c	5	4	N.D.	5.67	3.00	N.D.	4.00
C10	1	c	8	3	12.67	12.00	4.00	N.D.	10.67
C11	1	c	2	8	4.33	5.67	4.33	1.00	4.00
	2	c	1	8	10.00	9.00	6.33	3.67	7.67
	3	c	1	8	10.33	8.67	6.33	3.67	7.00
C12	1	e	7	6	4.67	6.00	5.00	1.67	4.00
	2	e	7	6	2.00	4.00	3.33	N.D.	3.00
	3	e	19	6	3.33	3.67	3.67	N.D.	4.33
C13	1	c	11	8	7.67	8.67	8.00	6.33	6.67
	2	c	11	8	9.33	8.67	6.00	3.67	6.00
	3	c	11	8	7.33	7.00	5.67	3.67	5.67
C14	1	c	23	6	2.50	4.00	3.75	0.75	3.00
	2	c	23	5	N.D.	4.33	N.D.	N.D.	3.00
	3	c	23	5	N.D.	3.67	N.D.	N.D.	3.00
C15	1	c/k	24	3	10.75	18.00	4.50	1.50	11.75
	2	c	21	7	6.50	7.25	6.00	4.00	6.00
C16	1	c	6	1	10.33	11.33	10.00	9.00	11.33
	2	c	6	6	12.33	10.33	7.33	4.33	11.33
	3	c	6	6	11.33	10.67	8.33	5.00	11.33
C17	1	c	3	7	8.33	8.67	7.67	5.00	6.67
	2	c/k	3	8	7.00	7.00	5.33	3.67	6.00
	3	c	3	8	6.67	7.33	5.33	3.67	6.33
UA159		c	9	4	13.00	12.67	4.00	N.D.	9.67
NBRC13955		c	12	4	17.33	22.67	7.00	N.D.	17.33

Darker red in the heat map indicates a larger inhibition zone. ‘N.D.’ indicates not detected.

**Table 2:** Characteristics and distribution of mutacin genotypes in 46 clinical isolates.

Mutacin Genotype	Positive Mutacin Genes	Number of Strains
1	*mukA, smbAB, nlmABCD*	2
2	*mukA, nlmABCD*	1
3	*smbAB, nlmABCD*	8
4	*nlmABCD*	12
5	*nlmCD*	2
6	*smbAB, nlmCD*	9
7	*mukA, smbAB, nlmCD*	2
8	*mukA, nlmCD*	8
9	*muk, nlmABC*	2

**Table 3:** Effect of nattokinase on mutacin activity.

Patient No.	Isolation No.	Mutacin Activity to Commensal Oral Bacteria (mm)				
		*S. sanguinis*	*S. gordonii*	*S. mitis*	*S. sobrinus*	*S. salivarius*
C1	1	9.00	7.33	2.00	N.T.	6.00
	2	1.00	3.33	2.00	N.T.	2.67
	3	0.00	3.00	3.33	N.T.	3.33
C2	1	3.33	3.33	3.33	N.T.	4.00
	2	2.00	3.33	3.33	0.00	3.67
	3	3.00	3.33	3.33	N.T.	3.67
C3	1	2.33	3.33	3.00	N.T.	5.33
	2	N.T.	3.33	3.33	N.T.	4.33
	3	1.00	4.67	2.00	N.T.	7.33
C4	1	20.00	10.00	8.67	4.33	9.00
	2	16.33	9.67	9.67	5.67	8.33
	3	11.33	10.00	9.00	6.67	7.33
C5	1	4.00	5.67	3.00	0.00	4.00
	2	4.00	4.33	3.00	N.T.	3.67
	3	6.00	6.00	2.00	N.T.	5.00
C6	1	1.50	3.00	3.33	N.T.	2.00
C7	1	2.00	3.67	3.67	0.00	2.00
	2	1.00	3.33	2.00	N.T.	2.00
	3	N.T.	3.00	3.00	N.T.	2.00
C8	1	8.67	9.00	8.67	7.00	10.67
	2	13.00	9.20	9.00	4.27	10.33
	3	14.10	9.50	9.30	4.97	10.67
C9	1	N.T.	4.67	2.00	N.T.	4.33
	2	N.T.	4.67	2.00	N.T.	4.33
	3	N.T.	4.67	3.00	N.T.	4.33
C10	1	7.00	6.33	4.00	N.T.	5.67
C11	1	3.67	5.00	4.00	1.00	3.67
	2	9.00	8.33	6.33	4.00	7.00
	3	10.00	8.33	6.33	4.00	6.67
C12	1	3.00	4.67	4.33	2.00	4.33
	2	2.33	3.33	3.33	N.T.	3.00
	3	3.33	3.33	3.33	N.T.	3.00
C13	1	6.33	7.67	6.33	5.33	5.33
	2	8.67	9.00	6.00	4.00	6.33
	3	8.33	9.00	6.33	4.33	5.00
C14	1	1.50	3.25	3.75	0.00	1.75
	2	2.00	3.00	N.T.	N.T.	2.00
	3	N.T.	3.33	3.33	N.T.	3.00
C15	1	5.25	7.75	4.00	0.75	6.50
	2	4.25	5.75	5.75	3.75	4.75
C16	1	10.33	10.00	9.33	7.33	9.33
	2	11.33	10.33	8.67	4.67	10.00
	3	10.67	9.67	8.00	4.67	9.33
C17	1	9.00	8.33	6.67	5.33	5.33
	2	7.67	7.67	5.33	4.00	5.67
	3	7.00	8.30	5.80	3.25	5.67
UA159		6.67	5.33	4.00	N.T.	6.00
NBRC13955		10.67	10.33	4.67	N.T.	11.67

‘N.T.’ indicates not tested.

**Table 4:** Effect of nattokinase on mutacin activity according to mutacin genotype.

Mutacin Gene	Number of Positive/Negative Strains for Mutacin Gene	Average of Mutacin Activity against Commensal Oral Bacteria *				
		*S. sanguinis*	*S. gordonii*	*S. mitis*	*S. sobrinus*	*S. salivarius*
*nlmAB*	**Positive (n = 27)**	**0.70**	**0.73**	**0.89**	**0.58**	**0.83**
	single (n = 14)	0.68	0.74	0.87	0.00	0.94
	+*smbAB* (n = 8)	0.57	0.61	0.85	0.17	0.57
	+*mukA* (n = 3)	1.03	0.93	1.03	1.10	1.07
	+*mukA*+*smbAB* (n = 2)	0.82	0.82	0.92	0.81	0.71
	**Negative (n = 21)**	**0.88**	**0.90**	**0.99**	**0.96**	**0.85**
*mukA*	**Positive (n = 15)**	**0.95**	**0.96**	**0.98**	**1.03**	**0.91**
	single (n = 8)	0.97	1.02	0.99	1.04	0.92
	+*smbAB* (n = 2)	0.87	0.88	0.91	1.00	0.80
	**Negative (n = 33)**	**0.70**	**0.74**	**0.91**	**0.60**	**0.80**
*smbAB*	**Positive (n = 21)**	**0.75**	**0.75**	**0.93**	**0.72**	**0.70**
	**Negative (n = 27)**	**0.83**	**0.85**	**0.93**	**0.97**	**0.94**

* Mutacin activity following the addition of nattokinase is expressed as relative value with no addition as 1.

## Data Availability

Related data and methods are presented in this paper. Additional inquiries should be addressed to the corresponding author.

## Supplementary Materials

The following supporting information can be downloaded at https://www.mdpi.com/article/10.3390/pathogens13040286/s1: Figure S1: Electrophoresis of AP-PCR analysis. Figure S2: Phylogenetic trees based on AP-PCR analysis and mutacin genotype; Figure S3: Effect of nattokinase on *S. mutans* biofilm formation; Figure S4: Spot assay conducted using agar medium with CSP addition; Figure S5: Effect of nattokinase on crude mutacin fraction; Table S1: Oligonucleotide primers used in this study and the amplicon size.
