# Supplementary material for: Nattokinase, a Subtilisin-like Alkaline-Serine Protease, Reduces Mutacin Activity by Inactivating the Competence-Stimulating Peptide in Streptococcus mutans
Source: Pathogens. 2024 Mar 27;13(4):286. doi: 10.3390/pathogens13040286 (PMC11054032; doi:10.3390/pathogens13040286)
Supplement: Supplementary file 1 [file pathogens-13-00286-s001.zip › pathogens-2893551-supplementary.pdf]

Supplementary Materials

Table S1. Oligonucleotide primers used in this study and the amplicon size.

Primer	Sequence (5'→3')	Amplicon	Length (bp)	Accession No.	Reference
**Bacteriocin Identification**					
mutI-F	GAAGTCCTTGGTACTGAAAC	*mutA* (mutacinI)	142	AF238860	This study
mutI-R	ACCCCTGTACATCCTAATGAAC				
mutII-F	GAACAAGTTAAACAGTAACGC	*mutA* (mutacin II)	136	U40620	This study
mutII-R	GCCATGAATTCATGCGACAC				
mutIII-F	TTATTAGAAGTCCTTGGTAC	*mutA'* (mutacinIII)	151	AF238860.1	This study
mutIII-R	GAGATCTTTCGAGGAGGGC				
mukA-F	AAGGAAGATACAACAACTAC	*mukA1-A3*	544	EF060238.1	This study
mukA-R	CATGAGTTGTAACGGCATTC				
smbA-F	TATGGTAACGCTCATCAAAG	*smbAB*	331	AB179778	This study
smbB-R	TGCATTCAACAGTAACCGTAC				
mutacinIV F(nlmA)	TGGATACACAGGCATTTGAAC	*nlmAB*	440	AE014133	This study
mutacinIV R(nlmB)	GGAAAAACTACAGATCCAAC				
mutV-F	GAATACACAAGCATTTGAAC	*nlmC*	227	AE014133	This study
mutV-R	TAACCACAGGAATTAAGAGC				
mutVI-F	GAATACACAAGCATTTGAAC	*nlmD*	227	AE014133	This study
mutVI-R	TCAACAAAAAGTAGCAGCAC				
**Quantitavite real-time PCR**					
nlmA F-RT	TGGATACACAGGCATTTGAAC	*nlmA*	109	AE014133	This study
nlmA R-RT	CATATTCCAATTGCCGCGAC				
nlmB F-RT	GCTGATACGTTTCTTTCAGC	*nlmB*	130	AE014133	This study
nlmB R-RT	GGAAAAACTACAGATCCAAC				
nlmC F-RT	GAATACACAAGCATTTGAAC	*nlmC*	88	AE014133	This study
nlmC R-RT	GCACCTAGAGCAATACCTGC				
nlmD F-RT	GAATACACAAGCATTTGAAC	*nlmD*	157	AE014133	This study
nlmD R-RT	GACTGGGAGAGTAACTGTAC				
16S F-RT	TGCCGAAGATTCCCTACTGC	16S rRNA	111	AE014133	This study
16S R-RT	AAGAGCTTACCAAGGCGACG				

**Construction of mutant**					
comCu-F	ccccgaattcACAGGAATTAAGAGCTCCTCC	*comC*	564	AE014133	This study
comCu-R	ccccggtaccCGCCAATGATAATCTCTAATTCATC				
comCd-F	ccccaagcttCAACATTTTTCCGGCTGTTTAAC	*comC*	522	AE014133	This study
comCd-R	cccctctagaACTGCCTGAGATGGAGTTGC				

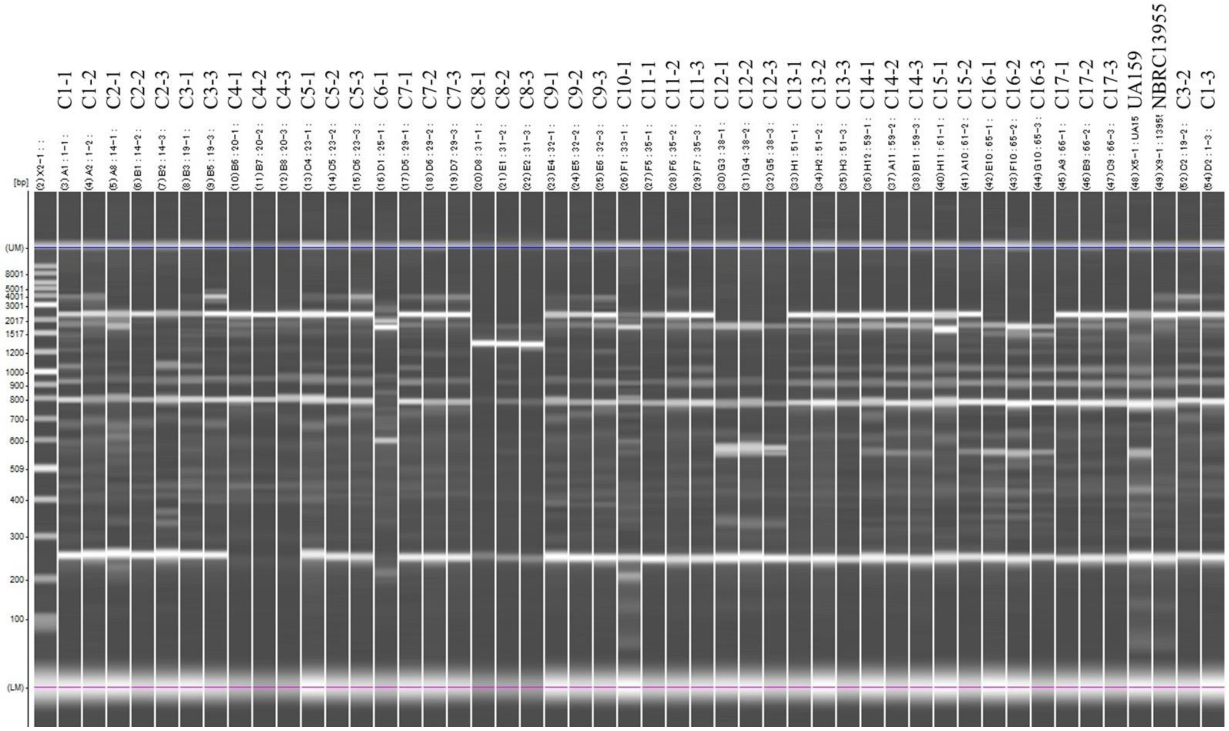

Figure S1. Electrophoresis of AP-PCR analysis.

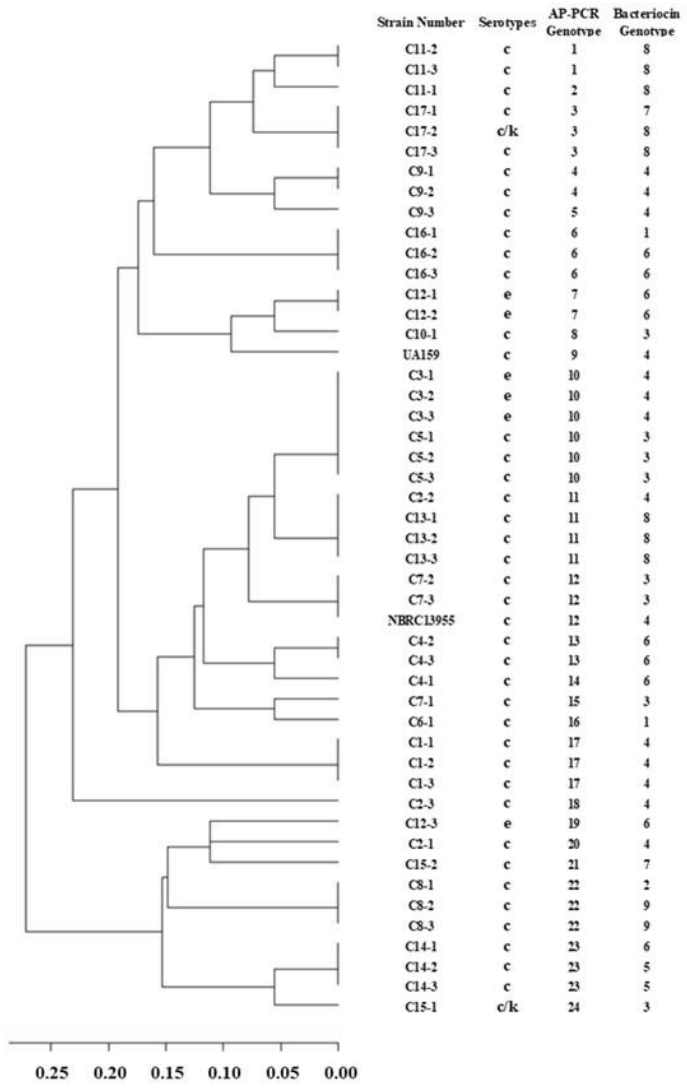

Figure S2. Phylogenetic trees based on AP-PCR analysis and mutacin genotype.

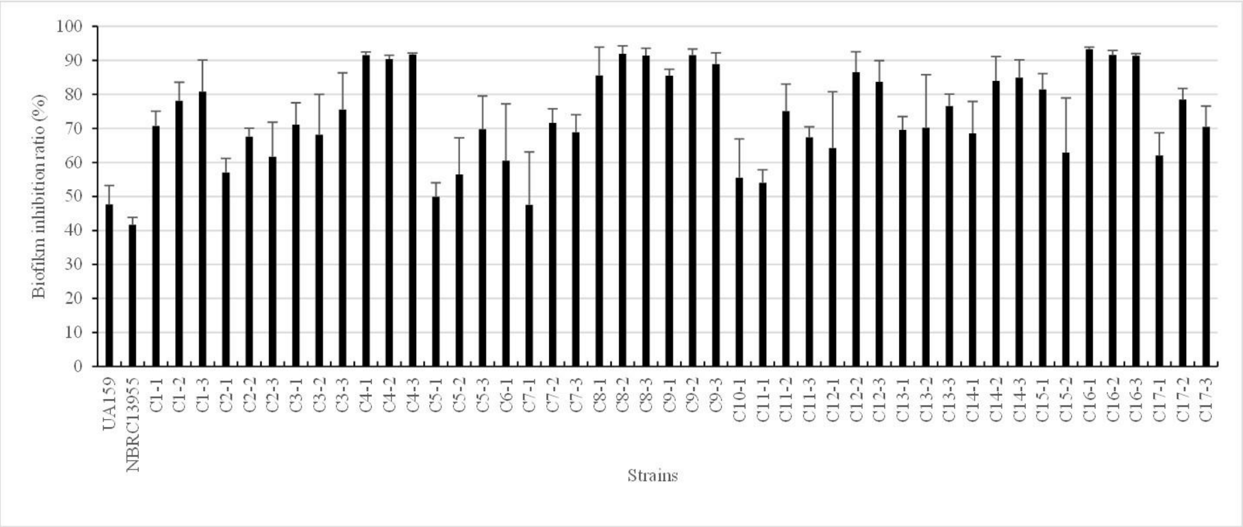

Figure S3. Effect of nattokinase on *S. mutans* biofilm formation.

Human saliva was used to assess biofilm volume on the saliva-coated surfaces. The pooled normal saliva collected from ≥ 3 donors (Cosmo Bio Co. Ltd., Tokyo, Japan) was clarified via centrifugation at 4,000 × *g* and 4 °C for 10 min. The supernatant was then filtered through a 0.22 µm pore filter and frozen at −20 °C until use. Aliquots (20 µL) of clarified saliva were added to 96-well polystyrene microtiter plates (Sumitomo Bakelite, Tokyo, Japan) and incubated at 4 °C for 20 h. The saliva was then decanted, and the wells were washed twice with phosphate-buffered saline (pH 7.4). Bacterial cells were collected via centrifugation at 8,000 × *g* and 4 °C for 10 min and resuspended in sterile distilled water to obtain an A_{660} = 0.5. *S. mutans* culture aliquots (2 µL) and 100 µL of 2 × tryptic soy broth without dextrose (TSB) medium (Becton Dickinson and Company, San Jose, CA, USA) and supplemented with 0.5% sucrose were added to microtiter plates, followed by the addition of nattokinase or sterile distilled water to a volume of 200 µL. The plates were incubated at 37 °C in a 5% CO_2 atmosphere for 20 h. After incubation, the wells of microtiter plates were washed with distilled water, and adherent cells were stained with 0.1% crystal violet. The dye was solubilized in acetate, and the A_{590} was determined using a microplate reader (Colona Electric, Ibaraki, Japan). The inhibition ratio (%) is expressed as relative values, with the control set as “100”. Data are expressed as the mean ± standard deviation of values from three independent experiments.

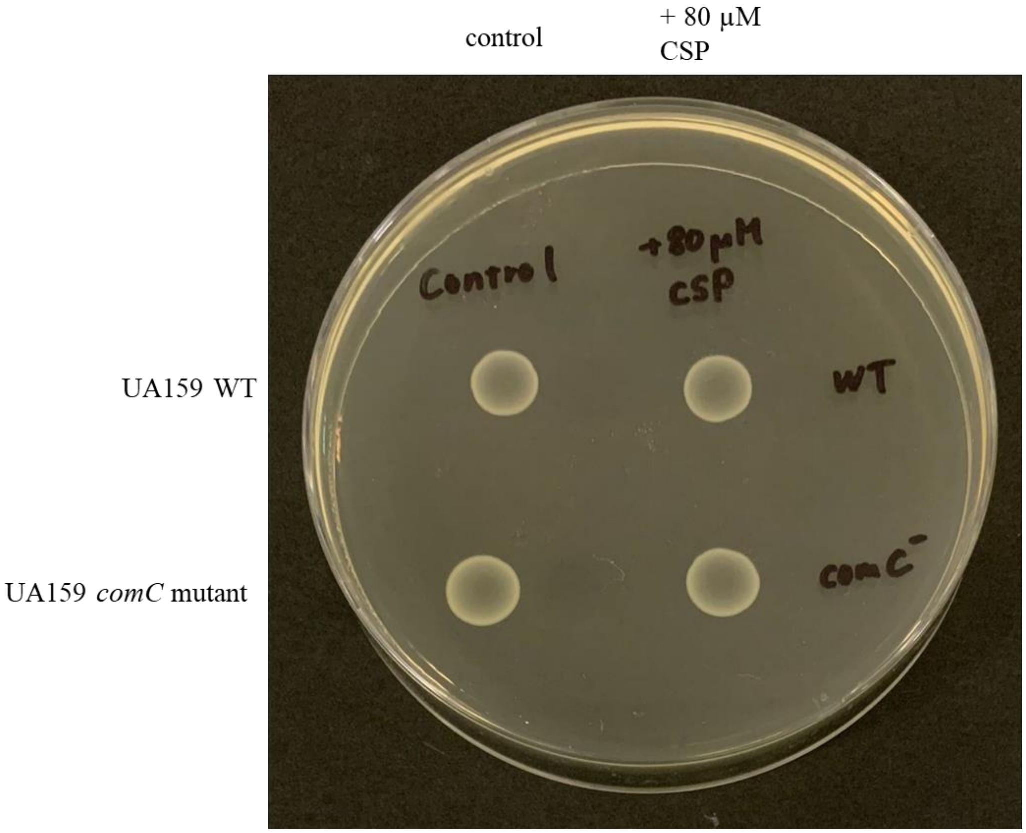

Figure S4. Spot assay conducted using agar medium with CSP addition.

CSP was prepared at a final concentration of 80 µM and mixed with *S. mutans* UA159 WT and UA159 *comC* mutant bacterial suspensions. Thereafter, 10 µL of the mixture was spotted on BHI agar and incubated at 37 °C in a 5% CO_2 atmosphere for 44 h. The growth inhibitory effect of CSP was assessed from the colonies formed.

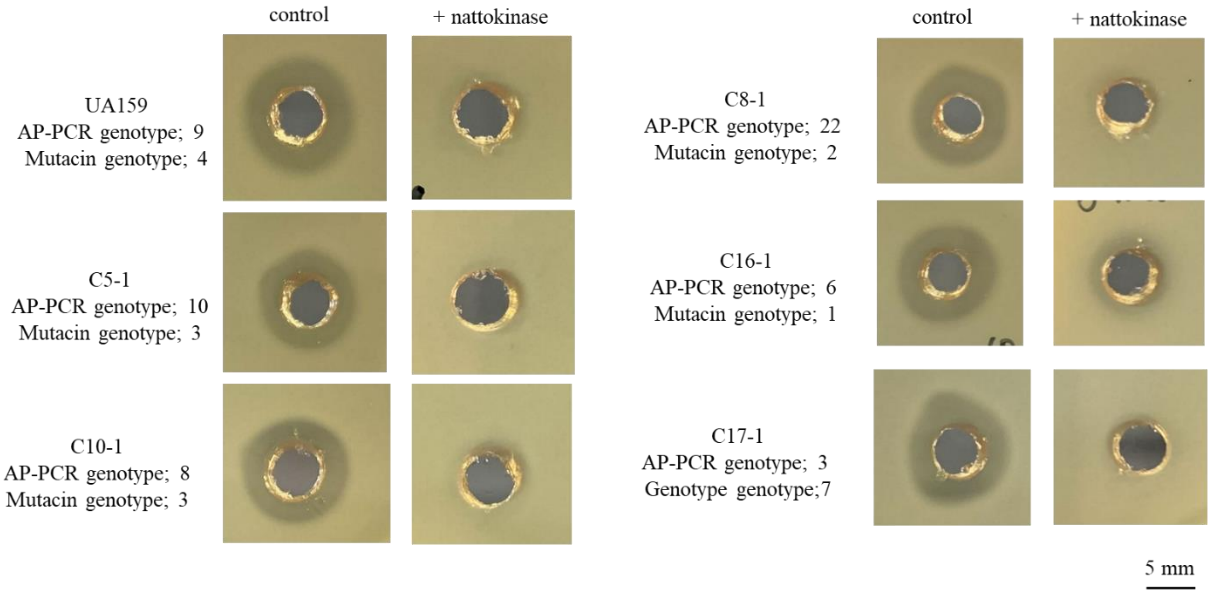

Figure S5. Effect of nattokinase on crude mutacin fraction.

A culture of *S. mutans* (300 µL) was inoculated into 30 mL TSB medium containing 0.13% (w/v) glucose and 6% (w/v) yeast extract. The mixture was incubated at 37 °C in a 5% CO_2 atmosphere for 20 h. After incubation, the culture was centrifuged at 8,000 × *g* and 4 °C for 10 min. The supernatant was then salted at 4 °C for 3 h via ammonium sulphate precipitation at an 80% saturation. After salting, centrifugation was performed at 9,390 × *g* and 4 °C for 30 min, and the precipitated fraction was dissolved in 1,500 µL of 0.1 M PBS (pH 7.4) (Fujifilm Wako Pure Chemical) to obtain a 20-fold concentration. The solution was sterilized via filtration through a 0.22 µm filter to give a crude purified mutacin solution. BHI agar containing 1% (v/v) *S. gordonii* DL1 culture was prepared, and 5 mm diameter holes were made. Then, 50 µL of crude purified mutacin solution was mixed with 5 µL of 10 mg/mL nattokinase or sterile water. Next, 50 µL of the mixture was added to the holes made in the agar. The medium was incubated at 37 °C in a 5% CO_2 atmosphere for 44 h. After incubation, the size of the inhibition circles formed was measured.
